# Characterizing the Substance Use Prevention Funding Landscape in the United States: a Cross-Sectional Study of National Prevention Network Representatives and Practitioners

**DOI:** 10.1007/s11121-026-01889-0

**Published:** 2026-03-10

**Authors:** Shirley S. Liu, Elvira Elek, Natalie Blackburn, Feker Wondimagegnehu, Parissa J. Ballard, Phillip W. Graham

**Affiliations:** 1 RTI International, 701 13th Street NW, Suite 750, Washington, DC 20005-3967 USA; 2 RTI International, Durham, NC USA; 3 NOVA National School of Public Health, Comprehensive Health Research Centre, NOVA University Lisbon, Lisbon, Portugal; 4 Wake Forest University School of Medicine, Winston-Salem, NC USA

**Keywords:** Substance use prevention, Implementation, Funding, Evidence-based interventions, Prevention infrastructure, Dissemination

## Abstract

Youth and adolescent substance use remains a persistent public health challenge in the United States; the delivery of evidence-based interventions (EBIs) is critical to improving related negative consequences. The Substance Abuse and Mental Health Services Administration funds a large portion of the implemented substance use prevention interventions in the United States by supporting a funding infrastructure that plays an important role in the adoption and scaling of interventions. Prevention intervention developers and researchers need to understand this infrastructure and its influence on local practitioners to increase the adoption of their EBIs. This study sought to identify which agencies in each state and jurisdiction are involved in funding allocation, how they prioritize and distribute funding to intervention implementers, and, subsequently, how they guide the selection of EBIs. This study used a mixed-methods, cross-sectional design to understand the infrastructure of prevention funding that underlies EBI decision-making. In 2023, we conducted surveys with 40 National Prevention Network representatives (NPNs) and 222 community-level practitioners; in early 2024, we conducted qualitative interviews with a subset of 16 NPNs. NPNs’ priorities were shaped by the agencies in which they were housed and the partners with whom they collaborated. Most were located within their state’s or jurisdiction’s department of health or behavioral health, and many engaged in partnerships with departments of public health or education. Most NPNs reported that they prioritized school and health settings and youth populations for prevention intervention delivery. Almost all NPNs directly distributed funding to intervention implementers (community, regional, or state entities); about half distributed some funds through an intermediary that then subcontracted another entity to implement interventions. More NPNs required or recommended that funded recipients select EBIs from lists or registries (75%) than required or recommended a specific strategy for at least some of their programs (53%). Many practitioners (47%) reported that they selected a recent strategy from a list of interventions provided by their funder, but 27% received no funder guidance on intervention selection. Prevention developers and researchers could increase the adoption of EBIs by focusing them on the priority areas for NPNs, including the health, behavioral health, and education sectors. Developers need to get their EBIs onto registries or intervention lists and increase the EBIs’ wide-scale dissemination. Audiences for information about specific EBIs should include NPNs, regional entities, and their funded community practitioner recipients.

## Introduction

Substance use among adolescents and young adults remains a concern in the United States. In 2022, 8.7% of adolescents (aged 12–17) and 27.8% of young adults (aged 18–25) could be classified as having a substance use disorder (Substance Abuse and Mental Health Services Administration, 2023a). Extensive research demonstrates the strong association between the early initiation of substance use in adolescence and the development of psychosocial problems, substance use disorders, and mental health disorders later in adulthood (Gobbi et al., 2019; Jordan & Andersen, 2017). Even as 2023 Monitoring the Future data show stable or declining rates of substance use among adolescents since the COVID-19 pandemic (National Institute on Drug Abuse, 2023), efforts to delay or prevent the onset of substance use and misuse remain critical to reducing later consequences. As such, evidence-based prevention efforts form an integral component of the continuum of care (Cance et al., 2023).

Despite the existence of a robust number of evidence-based interventions (EBIs), most programs and policies implemented across public systems are not EBIs (Fagan et al., 2019). Adolescent-focused interventions targeting some substances, such as alcohol, have been heavily tested and disseminated, but fewer EBIs exist to address other substances such as opioids. EBI development and dissemination must also respond to a rapidly evolving landscape of substance use: State cannabis policies have become increasingly less restrictive, though not uniform (Bryan, 2024), and trends in youth opioid overdoses show a rise in polysubstance use (Buchholz et al., 2025). In addition, although implementation science researchers widely agree on the importance of deimplementing nonevidence-based interventions to improve population health and optimize the use of resources (Norton & Chambers, 2020), Purtle et al. (2021) found that this activity was a low priority among policymakers. A better understanding by prevention program developers and researchers of the infrastructure that supports the selection and implementation of substance use prevention interventions could serve to increase the proportion of implemented EBIs.

This paper defines “substance use prevention infrastructure” as the financial resources and organizational framework in place to address substance use at the national, state, and local levels. This infrastructure supports all types of interventions, from individual prevention programs to community-based processes to environmental strategies that change the conditions that influence substance use (Substance Abuse & Mental Health Services Administration, 2020). In the United States, the state and local substance use prevention infrastructures play a pivotal role in the adoption, dissemination, implementation, and scaling of substance use prevention programs. Yet, in our experience working with both intervention developers and members of the state and local prevention infrastructure, many prevention researchers and developers have limited insight into how state and jurisdiction prevention systems operate, including how they distribute funding, support implementation, and facilitate the use of EBIs.

In the United States, the Substance Abuse and Mental Health Services Administration (SAMHSA) directly or indirectly funds a large portion of the implemented prevention interventions (National Academies of Sciences Engineering & Medicine, 2025; National Association of State Alcohol and Drug Abuse Directors, 2021). Each fiscal year, SAMHSA distributes an estimated $1.9 billion through the Substance Use Prevention, Treatment, and Recovery Services (SUPTRS) Block Grant funding mechanism (Substance Abuse and Mental Health Services Administration, 2023b). On average, these funds account for 62% of state primary prevention expenditure (National Association of State Alcohol and Drug Abuse Directors, 2021). Each state and jurisdiction in the United States receives SUPTRS funds through a lead agency, known as the Single State Agency (SSA). In addition, SSAs often competitively apply for discretionary grant funding from SAMHSA and other agencies. These funding mechanisms have more specific parameters guiding support for additional substance use prevention activities. At SAMHSA, discretionary grants include the Strategic Prevention Framework—Partnerships for Success (SPF-PFS) program; examples from other federal agencies include the Centers for Disease Control and Prevention’s Overdose Data to Action and the Office of National Drug Control Policy’s (ONDCP) Drug-Free Communities (DFC) grants.

Each SSA employs a National Prevention Network (NPN) director, who oversees substance use prevention-related funding at the state, tribal, or territorial level (Fishbein & Sloboda, 2022). Each SSA is governed by different statutes and operates using distinct organizational structures and funding distribution pathways that are shaped by its own jurisdiction’s governmental framework. However, all SSAs fund community providers to implement at least some of their substance use prevention interventions (Knopf, 2019).

The objective of this study was to understand the substance use prevention funding and EBI decision-making infrastructure at the state and local levels through two key groups of decision-makers within prevention systems: NPN representatives (NPNs) and community-level prevention practitioners. Four research questions shaped the data collection and analyses: (1) What agencies are involved in the substance use prevention funding allocation in states, territories, or jurisdictions? (2) What systems, populations, and settings do they prioritize? (3) What pathways exist to distribute funding to prevention implementers? (4) How do funders and others guide the selection of EBIs? Ultimately, this study aims to increase understanding of the prevention system among intervention developers, researchers, and research funders to capitalize on its potential for fostering both wide-scale EBI adoption and collaboration between developers, funders, SSAs, and community practitioners.

## Methods

### Study Design

This study followed an explanatory mixed-methods, cross-sectional design and used surveys to inform in-depth interviews. The surveys elicited information from two groups—NPNs and community-level prevention practitioners—on SSAs’ funding mechanisms and priorities, practitioners’ funding sources, and downstream guidance on intervention selection. The interviews engaged a subset of the surveyed NPNs and allowed for clarification and an improved understanding of the survey findings.

### Participants

NPNs operate as the prevention lead in each state’s, jurisdiction’s, or territory’s SSA, and they focus on supporting the implementation of effective, evidence-based prevention programs, practices, and policies related to tobacco, alcohol, and other substance use. NPNs oversee their jurisdiction’s substance use prevention systems, including the funding mechanisms that support prevention activities. The sample included 40 NPNs representing states or jurisdictions in each of the 10 regions delineated by the US Department of Health and Human Services (HHS; Table 1). The majority of the participating NPNs indicated their race as White (63%), their ethnicity as non-Hispanic (90%), and their gender as female (80%). Most indicated that they have substantial experience in both their current department or organization (about 88% had at least 3 years of experience) and in the prevention field in general (about 78% had at least 6 years of experience). Demographics of the 16 NPN interviewees reflected those of the NPN survey respondents.

**Table 1:** Demographics of survey participants

Variable	National Prevention Network representatives (*N* = 40)	Community prevention practitioners (*N* = 222)
	***N*** (%)	***N*** (%)
*Race*		
White	25 (62.5)	171 (77.4)
Black, African American	6 (15.0)	25 (11.3)
American Indian, Alaska Native	1 (2.5)	16 (7.2)
Native Hawaiian, Pacific Islander	2 (5.0)	3 (1.4)
Asian	3 (7.5)	4 (1.8)
Some other race	1 (2.5)	3 (1.4)
*Ethnicity*		
Hispanic	3 (7.5)	18 (8.1)
Not Hispanic	36 (90.0)	202 (91.4)
*Gender*		
Man	7 (17.5)	40 (18.1)
Woman	32 (80.0)	177 (80.1)
Other	0	2 (0.9)
*Years worked in current department/organization*		
Less than a year	2 (5.0)	27 (12.2)
1–2 years	3 (7.5)	38 (17.2)
3–5 years	13 (32.5)	40 (18.1)
6–10 years	9 (22.5)	43 (19.5)
More than 10 years	13 (32.5)	73 (33.0)
*Years worked in substance use prevention*		
Less than a year	1 (2.5)	19 (8.6)
1–2 years	1 (2.5)	28 (12.7)
3–5 years	7 (17.5)	24 (10.9)
6–10 years	8 (20.0)	44 (19.9)
More than 10 years	23 (57.5)	106 (48.0)
*HHS region*		
Region 1	5 (12.5)	23 (10.4)
Region 2	1 (2.5)	9 (4.1)
Region 3	6 (15.0)	19 (8.6)
Region 4	6 (15.0)	44 (19.8)
Region 5	3 (7.5)	18 (8.1)
Region 6	2 (5.0)	24 (10.8)
Region 7	3 (7.5)	20 (9.0)
Region 8	5 (12.5)	37 (16.7)
Region 9	7 (17.5)	14 (6.3)
Region 10	2 (5.0)	12 (5.4)

*HHS* US Department of Health and Human Services

Community-level prevention practitioners (hereafter referred to as “practitioners”) work at local organizations that receive prevention funds and have a role in decision-making around the adoption of prevention interventions and strategies in their communities. The 222 practitioner participants represented 45 states and jurisdictions across all 10 HHS regions (Table 1) and indicated that they worked in nonprofit community organizations (54%), coalitions (29%), and local government agencies (17%). They predominantly described themselves as White (77%), non-Hispanic (91%), and female (80%). These practitioners identified themselves primarily as project coordinators (31%), project directors (30%), and organizational directors (22%). Most had at least 3 years of experience in their organization (71%) and at least 6 years of experience in the prevention field (68%).

### Procedures

A former NPN provided a comprehensive list of all current state, jurisdiction, and territorial NPNs (*N* = 55) and their contact information. For NPN survey recruitment, the NPNs were sent a personalized email, which included a unique REDCap-generated survey link. The initial attempt was followed by up to four rounds of email messages and telephone contacts. The recruitment emails requested that the NPNs participate in a study on substance use prevention funding and decision-making infrastructure and offered them a $15 Amazon gift card as an incentive. We sought to conduct follow-up interviews with 25% of those surveyed; this goal reflected available resources and best practices for achieving thematic saturation (Squire et al., 2024). We purposively selected interview participants to ensure geographic diversity across US regions. Recruitment for the interviews consisted of an email outreach that requested participation in a 45-min interview to follow up on survey responses. In cases of nonresponse, the recruitment process involved follow-up attempts and the inclusion of alternates to meet our sampling goal. Each interview took approximately 1 h, and all took place during March–May 2024.

We recruited practitioners through three processes: (1) referrals from NPN representatives who participated in the survey (28%), (2) in-person recruitment through a booth during the 2023 NPN conference (45%), and (3) an informational email sent to all attendees of the 2023 NPN conference (28%). The annual NPN conference brings together prevention professionals, government partners, and researchers to share substance use prevention best practices based on research and evaluation findings (National Association of State Alcohol and Drug Abuse Directors, 2025). Participants at the conference completed the survey on tablets or on paper forms. Other practitioner participants received a personalized email with a unique survey link that sent them to a REDCap-generated survey form; the initial request was followed by up to four rounds of email and telephone follow-up. The in-person conference recruitment pitch and the emails asked potential participants to complete a survey and offered them a $15 Amazon gift card as an incentive. To participate in the survey, practitioners had to be knowledgeable about the resources their organizations received and used to fund substance use prevention activities and the ways in which their organizations selected strategies or interventions to implement the activities in their communities. The survey invitation allowed recipients to recommend an alternative respondent from their organization if they felt another person was better suited to complete the survey. Practitioners completed their surveys from August through December 2023.

### Instruments

The survey instruments were developed through expert input, pilot testing with both NPNs and practitioners, and cognitive interviews with practitioner representatives. The semistructured interview guides built upon the surveys to allow for further elaboration and understanding of responses. Questions from the survey and interviews focused on interagency collaborations in prevention funding, respondents’ areas of focus for funding (systems, populations, and settings), pathways used by NPNs to fund substance use prevention, and the funding sources used by practitioners to support their prevention activities. NPNs also provided information on how they provided guidance on decisions about selected EBIs, including which other entities participated in that decision-making. The practitioner survey also asked questions about the guidance practitioners received in making decisions about selecting interventions.

The Office of Research Protection at RTI International deemed this study exempt from human subjects research under Category (2)(ii) of the Revised Common Rule (#STUDY00022136).

### Analysis

We used Stata 18 software to conduct the quantitative analysis. For demographic variables, we summarized information to provide details on the characteristics of the sample using frequencies, means, and standard deviation. Basic frequencies and percentages summarize the findings from the surveys.

Qualitative analyses of the interviews, which used a sequential explanatory design, helped explain the survey results. Interviews were audio-recorded, deidentified, transcribed verbatim, and imported into NVivo 12. We undertook a deductive–inductive analysis approach, using the Consolidated Framework for Implementation Research to develop an initial codebook for content analysis and additional codes identified as relevant (Damschroder et al., 2022). Coders first reviewed three interview transcripts together to calibrate the codebook and ensure reliability among coders. The research team then aligned relevant codes from the qualitative data to explain the results of the quantitative data.

## Results

### Interagency Partnerships and Collaborations in Support of Substance Use Prevention

The agencies that NPNs are housed in and the other agencies they partner with may influence their priorities for substance use prevention, which then influences their interest in specific EBIs. About half of the responding NPNs worked within their state’s health, health care, or health and human services department (48%), whereas others worked in their state’s departments of mental health (28%), behavioral health (10%), or public health (8%). The remainder were housed in drug and alcohol departments, territorial behavioral health authorities, the state Medicaid agency, and a department of aging and disability.

To assess potential partnerships in substance use prevention, the survey asked NPNs to select the other agencies that funded substance use prevention in their state or jurisdiction. NPNs commonly selected the department of public health (83%) and the department of education (63%) in response to this question (see Table 2). In the interviews, NPNs elaborated on which other state agencies funded substance use prevention; they specifically mentioned collaborations with departments of public health, education, human services, and transportation. Such collaborations involved sharing data and coordinating their substance use prevention efforts, including, at times, multiple agencies’ contributing funding to specific prevention strategies. As an example of one such collaboration, one NPN stated,

The office of the Department of Law and Public Safety contacted us recently because they wanted to provide Deterra drug disposal kits to hospitals around the state. We were able to allocate funding from the block grant supplement to enable them to do that.... We don’t operate in isolation. But whatever we need to do to collaborate and most effectively meet the needs of the people in [State]—that’s what we try and do.

**Table 2:** Agencies in your state, territory, or jurisdiction that fund substance use prevention

Agency or department	*n* (%)
Public health	33 (82.5)
Education	25 (62.5)
Social services or child welfare	16 (40.0)
Mental health and developmental disabilities	11 (27.5)
Justice or corrections	11 (27.5)
Transportation	5 (12.5)
Housing	3 (7.5)
Other^a^	10 (25.0)
None	1 (2.5)
Do not know	2 (5.0)

Responses are those of National Prevention Network representatives (*N* = 40). Numbers may not add up to 100%, as response options were not mutually exclusive (select all that apply)

^a^Respondents who selected “Other” were asked to write in the agency; these responses included alcohol control boards, the attorney general’s office, and highway safety agencies

As noted, the majority of NPN survey respondents reported that substance use prevention funding also came through their department of education. During interviews, some NPNs described a long-standing partnership with their department of education, particularly through family and youth resource centers and student assistance programs to reach youth. Some NPNs also mentioned the key role of their department of education in epidemiological outcomes workgroups, jurisdiction-wide collaborative groups that enhance the use of data to drive substance use prevention strategies. In one state, the NPN even managed high school survey data for their department of education.

### Prioritized System, Population, and Setting

Where and to which populations a state or jurisdiction directs substance use prevention funds provides a clear indication of its priorities for supporting specific prevention activities and infrastructure. In their surveys, NPNs responded that their SSA commonly distributed funds to elementary, middle, and high schools (95%) and to outside-of-school youth program settings (93%). Furthermore, SSAs for 65% of the NPNs distribute at least some funds to health settings, including health departments, hospitals or other health care facilities, and emergency departments. Of the listed systems and settings, NPNs least often indicated that their SSA-distributed funds to the adult legal system (18%).

When asked to identify the populations that SSA-distributed prevention funds were focused on, NPNs most often reported that rural populations received this focus, with 85% of NPNs specifically directing prevention efforts toward this demographic. In addition, 85% of NPNs reported focusing on at least one specified racial or ethnic minority group. NPNs also prioritized individuals from underresourced communities (78%) and lesbian, gay, bisexual, transgender, queer, intersex, and asexual populations (65%). Of the listed groups, NPNs less often indicated that SSA funds were focused on individuals experiencing homelessness or on individuals without health insurance (28% and 25%, respectively).

Generally, the interviewed NPNs described a strong but not ubiquitous focus on reaching youth through school settings, along with more varied, community-specific approaches to reaching other populations. NPNs reported that they wanted to engage adults and parents as influencers in preventing youth substance use, as well as directly target older adults to prevent substance use in that population, but they faced barriers to finding the best place to reach them and securing enough funding to serve them. One NPN innovated by collaborating with local businesses to reach a wider range of people:

We’ve worked with a whole bunch of groups that I never thought we would work with.... One of my favorites is the funeral industry, because we were disseminating medication disposal kits. A lot of people would come in and their loved ones have passed—there’s a whole bunch of medicine in the medicine cabinet. What do they do with it? And so, some of our providers have been giving them instructions and chemical medication disposal kits as an example.

Another NPN mentioned doing some work in senior citizen centers to reach older adults but felt their work there was inconsistent.

When asked about their focus on rural populations, interviewees discussed how the rural nature of many areas posed challenges to service delivery because of the vast geographic spread and limited resources. One NPN expressed difficulty reaching parents via social media and virtual events in their rural state because many did not have access to high-speed internet. Another interviewee described a syringe exchange program in their state but noted that rural parts of the state had less access to it:

The [syringe] program is based in part of the county that’s maybe down here, and you still have all the state up here. So, our biggest conundrum is [most of the state’s] accessibility to all things related to substance use treatment, harm reduction, prevention.

NPNs also adapted their strategies to address the unique characteristics of rural populations.

### Funding Distribution Pathways

The survey asked NPNs how they distributed various types of funding—SUPTRS (block grant) funds, discretionary prevention funds, and other funds—and provided response options that illuminated decisions about direct or intermediary (through other state agencies or regional entities) funding pathways and the entities that received the funding to implement strategies. Table 3 displays the number of NPNs that reported distributing each type of fund through either direct or intermediary funding pathways. All NPNs reported distributing some block grant funds (100%), discretionary prevention funds (80%), or other funds (98%) through direct funding. Fewer NPNs reported distributing block grant funds (33%), discretionary prevention funds (28%), or other funds (40%) through an intermediary. Table 4 displays information on which entities ultimately received resources to implement the strategies funded by the SSAs. Although fewer than half of the NPNs reported implementation by staff within their own SSA (45%), most reported implementation by other state or regional organizations (93%) and community entities (93%).

**Table 3:** National Prevention Network representatives’ pathways to distribute funding

Type of pathway	Block grant funding *n* (%)	Discretionary funding *n* (%)	Other funding *n* (%)	Total *n* (%)
Direct	40 (100.0)	32 (80.0)	39 (97.5)	40 (100.0)
Through intermediary	13 (32.5)	11 (27.5)	16 (40.0)	19 (47.5)

Responses are those of National Prevention Network representatives (*N* = 40). Numbers may not add up to 100%, as response options were not mutually exclusive (select all that apply)

**Table 4:** Entity implementing Single State Agency–funded strategies

Implementer	Block grant funding *n* (%)	Discretionary funding *n* (%)	Other funding *n* (%)	Total *n* (%)
SSA staff	15 (37.5)	11 (27.5)	13 (32.5)	18 (45.0)
Other state agency, regional organization, or state-level contractor	32 (75.0)	23 (57.5)	33 (82.5)	37 (92.5)
Community entities	36 (90.0)	26 (65.0)	32 (80.0)	37 (92.5)
N/A (other)	13 (32.5)	11 (27.5)	16 (40.0)	19 (47.5)

Responses are those of National Prevention Network representatives (*N* = 40). Numbers may not add up to 100%, as response options were not mutually exclusive (select all that apply). *SSA* Single State Agency

NPNs reflected on the focus on community-level implementation in their interviews. A recurring theme was that funds went directly to communities or were used for mini local grants. Many NPNs specifically mentioned community coalitions as funding recipients. In addition to directing funds to community coalitions, NPNs described the use of funds for capacity-building initiatives at the state and local levels and for building community relationships with key organizations involved in prevention efforts. These relationships extended to such community organizations as housing authorities, homeless shelters, the funeral industry, real estate entities, and chambers of commerce. The connections of communities with key organizations allowed for targeted outreach and engagement with diverse populations.

The NPN interviews also provided more information on how much of the SUPTRS funding went directly to prevention efforts, rather than to treatment, recovery, or other activities. Most interviewees felt that they did not have adequate prevention resources, as NPNs consistently reported allocating no more than the 20% SAMHSA-required minimum prevention funding set-aside to prevention activities, although their agencies had the flexibility to increase that funding. However, one interviewee described a different way to allocate SUPTRS funds in their state. Because Medicaid covered some treatment services in their state, this NPN could use block grant funds that would typically go toward treatment for prevention instead. This bumped their prevention set-aside to 40%–45% of the SUPTRS, which they felt was sufficient. The NPN attributed this success in increasing their prevention allocation of SUPTRS funding to their ability to advocate for prevention and to leverage alternative payment models.

### Funding Sources or Pathways Reported by Practitioners

The practitioner surveys elicited information about the source of the funds that practitioners received to support their substance use prevention activities. When asked more generally about who directly funded their organization, agency, or coalition to support their strategy implementation, practitioners named state- or jurisdiction-level agencies (84%), the federal government (68%), regional entities within the state or jurisdiction (32%), and community coalitions (20%). Some practitioners also received funding from other sources, including foundations or nonprofits (36%), individual donations or fundraising events (29%), and corporate/business entities (17%).

A separate item asked practitioners to indicate whether their organization, agency, or coalition had received funds (either directly or indirectly) from a list of specific government-supported sources of funding (grants and programs) in the past 12 months. Among the sources of funding they received, practitioners also reported which sources they used to directly fund substance use prevention strategies. To fund substance use prevention activities, practitioners most commonly reported that their organizations received and used the SUPTRS Block Grant (47%) and SPF-PFS (46%), both supported by SAMHSA (see Table 5). Around a quarter of practitioners’ organizations received and used SAMHSA State or Tribal Opioid Response Grants (29%) and ONDCP DFC grants for prevention (25%). Of note, among these sources, only the DFC grants provide federal funding directly to community organizations; all other sources route federal funds through state or jurisdictional entities before they reach communities.

**Table 5:** Practitioner-reported direct and indirect funding sources

Funding source	Received by their organization and used to fund substance use prevention
	***n*** (%)^a^
Substance Abuse and Mental Health Services Administration (SAMHSA) Substance Use Prevention, Treatment, and Recovery Services (SUBG)	99 (46.5)
SAMHSA Strategic Prevention Framework Partnerships for Success (SPF-PFS)	97 (45.5)
SAMHSA State or Tribal Opioid Response Grants (SOR or TOR)	62 (29.1)
Office of National Drug Control Policy (ONDCP) Drug-Free Communities (DFC) Grant	53 (24.9)
Opioid settlement funds (from lawsuits directed at drug companies)	36 (16.9)
Synar (focused on sale and distribution of tobacco products to minors)	28 (13.1)
SAMHSA SPF for Prescription Drugs (SPF Rx)	27 (12.7)
SAMHSA Sober Truth on Preventing (STOP) Underage Drinking	24 (11.3)
SAMHSA State Targeted Response to the Opioid Crisis Grants (Opioid STR)	21 (9.9)
Centers for Disease Control and Prevention (CDC) Overdose Data to Action—States (OD2A-S)	17 (8.0)
SAMHSA Comprehensive Addiction and Recovery Act (CARA) Local Drug Crises Grants	15 (7.0)
SAMHSA Grants to Prevent Prescription Drug/Opioid Overdose-Related Deaths (PDO)	10 (4.7)
CDC OD2A—Local	10 (4.7)
Medicaid (federal, state, local)	6 (2.8)
SAMHSA Harm Reduction Program Grant	4 (1.9)
SAMHSA Medication-Assisted Treatment—Prescription Drug and Opioid Addiction (MAT-PDOA)	3 (1.4)
Other local government funds	34 (16)
Other state, tribal, or jurisdiction funds	29 (13.6)
Other SAMHSA funds	15 (7.0)
Other federal funds	15 (7.0)

Numbers may not add up to 100%, as response options were not mutually exclusive (select all that apply)

^a^*N* = 213 (9 missing)

In response to an open-ended question asking how they identify sources of funding to support substance use prevention strategies, practitioners frequently reported using government funding announcements, requests for proposals, government websites, and grants.gov. A few described word of mouth, networking, or contacts with local or community partners as facilitating their identification of grants. A small number of practitioners also listed private foundations, corporate donors, and businesses as sources of funding information. Notably, multiple practitioners described a process in which they sought out grants by first outlining their own goals and then searching for grants that aligned with those goals.

### Guidance for Selecting Evidence-Based Interventions

Table 6 presents information on how NPNs guide the decisions of the practitioners they fund to adopt particular prevention strategies or interventions. This table displays NPNs’ responses to a question asking them whether and how their SSA requires or recommends specific evidence-based substance use prevention strategies for at least some of their funded programs. Only 2 of the 40 responding NPNs did not issue any recommendations or requirements regarding their strategy selection. NPNs most commonly reported that they required practitioners to select evidence-based prevention strategies from a list or registry for at least some funded programs (58%). Requiring or recommending that practitioners choose from a list or registry was more common than requiring or recommending that they choose a specific strategy (75% compared to 53%). NPNs also reported that their SSAs tended to require rather than recommend strategies (75% compared to 55%), either through a list or registry or as a specific strategy. In their interviews, several NPNs noted challenges with using registries and lists, specifically in ensuring that the supportive research and information provided is up to date. One interviewee explained,

I think that the challenge, besides just maintaining [our repository] … is just ensuring that the resources are relevant to the times. Annually, … we meet to just kind of go over our list and make sure that it’s getting cross-referenced with any type of research or data that indicates that this resource has been deemed to maybe not be as effective as it was 10 to 15 years ago.

**Table 6:** National Prevention Network representatives’ reported guidance for strategy selection

Type of guidance provided	*n* (%)
We require specific evidence-based prevention strategies for at least some funded programs	14 (35.0)
We require practitioners to select evidence-based prevention strategies from a list/registry for at least some funded programs	23 (57.5)
We recommend specific evidence-based prevention strategies for at least some funded programs	13 (32.5)
We recommend practitioners select evidence-based prevention strategies from a list/registry for at least some funded programs	14 (35.0)
We do not recommend or require specific evidence-based prevention strategies or the selection of strategies from a list/registry for any of our funded programs	2 (5.0)

Responses are those of National Prevention Network representatives (*N* = 40). Numbers may not add up to 100%, as response options were not mutually exclusive (select all that apply)

Table 7 shows practitioners’ responses to a question on how their funder recently influenced their decision to select a substance use prevention intervention that was not required by the funder. Practitioners most frequently reported that they selected their strategy because it was on a list of interventions provided by their funder (47%). Fewer practitioners (16%) reported that they selected their strategy because their funder recommended that specific strategy. A substantial number of practitioners (27%) reported that they did not receive any guidance from their funder regarding intervention selection.

**Table 7:** Funder influence on practitioners’ recent substance use prevention intervention selection

Practitioner reason for selecting intervention	*n* (%)^a^
Our funder recommended it	35 (15.8)
It was on a list of interventions provided by our funder	103 (46.6)
We did not receive any guidance from our funder on intervention selection	59 (26.7)
Other	55 (24.9)

Numbers may not add up to 100%, as response options were not mutually exclusive (select all that apply)

^a^*N* = 221 (1 missing)

NPNs who indicated that their SSA provided some guidance on selecting substance use prevention strategies (*n* = 36)—either requiring or recommending specific strategies or ones from a list—were also asked what other entities were involved in this decision (see Table 8). Those involved included state-level evidence-based practice workgroups (reported by 56% of the NPNs who provided guidance) and state-level policymakers outside of the SSA office (17%). Some NPNs (42%) reported the involvement of other groups such as state staff, regional or local health authorities, community coalitions, and technical assistance providers.

**Table 8:** Entities involved in making decisions about recommending or requiring evidence-based prevention strategies

Entity	*n* (%)
State-level policymakers (individuals outside of the SSA office)	6 (16.7)
State-level evidence-based practice workgroups	20 (55.6)
Multistate regional workgroups	2 (5.6)
Tribal entities	1 (2.8)
Other	15 (41.7)
Do not know	1 (2.8)

Responses are those of National Prevention Network representatives (*N* = 36 [4 missing]). Numbers may not add up to 100%, as response options were not mutually exclusive (select all that apply). *SSA* Single State Agency

The NPN interviews provided more insights on this partner involvement in decisions about requiring or recommending strategies. The amount of guidance provided, collaboration between entities, and input from practitioners varied by state. In some instances, decision-making authority rested with state-level teams or individuals. These teams or individuals were often agency staff and workgroups that were responsible for reviewing and approving new strategies or evidence-based prevention programs. Although evidence-based practice workgroups played an important role in many states, the interviews highlighted struggles in establishing them, such as difficulties with member recruitment and financial constraints.

Some NPNs highlighted the importance of community-level input. Communities identified local needs, conducted needs assessments, and proposed strategies that were most suitable for their populations. State entities considered the insights and recommendations of local coalitions or organizations when reviewing proposals for new strategies or evidence-based prevention programs. For example, one NPN described relying on their regional entities in the state to understand local needs and then supporting the entities on the basis of that information. They described the process this way:

Our regions do a full strategic plan and needs assessment. So, they are telling us their logic model and the things that they see are the local contributing factors to their problems. And then we are trying to really match them up with the best practice for them.

Some interviewees noted that intervention selection was made at the community level and then sent up to the state NPNs for review, perhaps reflecting those practitioners who noted not receiving guidance from their funder on intervention selection. One NPN described review of a community-level decision as follows:

The determination regarding the program or strategy is made at the community level. But then, you know, we are able to review that decision when we review that application for funding. If we have any issues or concerns about that, maybe they won’t get funding. But we sometimes do require that they make adjustments to what they plan to do. But generally, what they propose, we find acceptable because they have been doing this work for so long. So we trust their judgment.

These interview responses show a bidirectional and collaborative relationship between state-level oversight and community-driven decision-making.

## Discussion

This descriptive portrait of the national substance use prevention infrastructure provided by NPN representatives and community-level practitioners in 2023 and 2024 details how agency partnerships, state-level priorities, and funding pathways might shape decision-making around the adoption of EBIs.

### Funded Agencies, Partnerships, and Priorities

Goldstein et al. (2023) describe SUPTRS funding as a major pathway for substance use prevention but note the variability in funding eligibility for prevention interventions across states. About two-thirds of the practitioners surveyed for this study relied on SUPTRS funding, and many used other federal sources of funding to support their substance use prevention activities. However, the influence of federal resources on EBI selection is limited by competing state and federal priorities, exposure to policy shifts, and the inherently time-limited nature of most funding cycles (National Academies of Sciences Engineering & Medicine, 2025). Findings from this study suggest that SSAs can play a substantial role in influencing which interventions get selected, and their agency affiliations, partnerships, and priorities may play a role in those decisions. Notably, health- or behavioral health-related agencies commonly housed SSAs and the NPNs participating in this study, and the education and public health sectors represented important partners and sources of support that extend the reach of substance use prevention initiatives.

The NPNs in this study reported prioritizing a focus on youth, consistent with prevention frameworks emphasizing early intervention (Kim et al., 2015; Rhew et al., 2013). Collaborations with education agencies allow SSAs access to schools where implementers can reach a large population of youth at once and incorporate programming into school curricula (Benningfield et al., 2015), and many EBIs already exist for school settings (Griffin & Botvin, 2010). The NPNs often also reported prioritizing rural communities, likely because of the higher rates of opioid use, fragmented health care, socioeconomic forces, and decreased access to services in these areas (Ezell et al., 2021). Fewer EBIs have been developed or adapted for rural populations, although tailored interventions for these populations may prove effective (Hecht et al., 2018).

Despite the potentially substantial need for services among older adults, people experiencing homelessness, and justice-involved populations, few of the surveyed NPNs reported funding programs for them, possibly indicating the NPNs’ reliance on other agencies and partnerships to reach these populations and address their unique needs and disparities (Bronson et al., 2017; Gaeta Gazzola et al., 2023; Han & Moore, 2018; Polcin, 2015). The National Institutes of Health (NIH) recognized these needs and gaps in its funding of the Helping to End Addiction Long-term, Preventing Opioid Use Disorder program (NIH, 2022), which supported the development of interventions for older adolescents and young adults involved in the justice system, experiencing homelessness, or encountering child welfare systems. Prevention intervention developers may also find promising opportunities to collaborate with NPNs and other state partners to disseminate existing interventions that reach these underserved populations.

### Funding Pathways

Funding source requirements (Substance Abuse and Mental Health Services Administration, n.d.) or state or jurisdiction policies often dictate whether SSAs can distribute funding directly to organizations to implement substance use prevention strategies or whether they need to fund intermediary organizations (other state agencies or regional entities), which then directly fund the organization implementing the prevention strategies. In addition, as the NPNs in this study shared, funding may go to implementation by SSA staff themselves, to contracted state agencies or regional organizations, or to community entities such as county, city, or tribal governments; community coalitions; or individual providers or organizations. An understanding of these funding pathways provides some guidance on which organizations might most directly influence decision-making about EBI adoption. Survey responses indicate a reliance on direct state or jurisdiction funding mechanisms; most of the surveyed practitioners reported receiving funds directly from those sources, and all surveyed NPNs reported allocating some funds directly to entities that implement strategies, bypassing intermediary organizations. This direct approach allows SSAs to have greater influence over the decision-making about what interventions implementers adopt. However, only about half of the surveyed NPNs also used an intermediary funding pathway, which suggests a potential underutilization of regional entities that could enhance coordination and resource sharing across more specified geographic areas. Ballard et al. (2021) and Piper et al. (2012) both highlighted the potential role of regional entities in providing oversight to direct prevention providers. Further research should explore what drives states to use more direct or intermediary pathways to distribute funding.

### Community Engagement in EBI Selection

Our study also found that NPNs placed an emphasis on community-level implementation and decision-making, underscoring a key opportunity for developers and researchers to integrate community perspectives into intervention design to improve relevance and increase the likelihood of EBI adoption. Nearly all the NPNs reported funding community coalitions and organizations to implement prevention strategies, signaling recognition of the value of localized, culturally tailored prevention approaches. The epidemiological outcomes and evidence-based practice workgroups described by the NPNs at the state level provide community practitioners with county-level and sometimes more localized data and information about relevant EBIs. Practitioners are encouraged to further explore local data through collaborating with local organizations (law enforcement, hospitals, schools) and gathering community input (e.g., through town halls). This helps them identify contextually salient risk and protective factors to strengthen the cultural relevance of their selected EBIs to their populations and improve their alignment with community-specific substance use profiles. However, although the NPNs in this study considered community involvement to be crucial to decision-making, their answers implied that community engagement was at the SSA’s discretion rather than thoroughly embedded in the prevention infrastructure. Enhancing community engagement mechanisms could strengthen the alignment between state-level priorities and local needs, optimizing the effectiveness of prevention efforts.

### Guidance and Registries for EBI Selection

We found that requiring the selection of EBIs from a list or registry was the most common form of guidance provided by state agencies, and more than half of the NPNs required or recommended the use of specific EBIs for at least some of their funded programs. This practice aligns with the Society for Prevention Research’s MAPS IV Translation Research Task Force in 2016, which identified several key factors affecting EBI scale-up, including policies that mandate or recommend EBIs; such mandates for EBIs significantly enhance their adoption and implementation in public systems (Fagan et al., 2019). We found that clearinghouses and registries were particularly important to both NPNs and practitioners in decisions to adopt EBIs. However, the variation in guidance and the noted challenges in maintaining up-to-date registries that reflect new evidence for different populations indicate a need for more robust and dynamic systems to support the continuous dissemination of evidence-based knowledge (National Academies of Sciences Engineering & Medicine, 2025). Other scientists in the federal space have recommended standardizing the use of registries and other intervention criteria to accelerate research into practice (Goldstein et al., 2023). Given the centrality of registries in EBI selection decisions, developers should prioritize getting their interventions on the registries and lists used by decision-makers, including registries such as Blueprints for Healthy Youth Development and state lists such as the state of Washington’s Excellence in Prevention page.

## Limitations

This study describes one substantial piece of the substance use prevention funding infrastructure in the United States, but the constitution of the NPN and practitioner samples may mean that some perspectives and unique funding pathways were missed. We successfully included about three-quarters of NPNs, and both the NPNs and practitioners represented a broad geography across the United States and its territories. However, we did not reach out to prevention funding administrators at other agencies (e.g., departments of education), and practitioner recruitment relied heavily on NPNs through referral and practitioner attendance at the NPN conference. This NPN-dependent sampling of practitioners likely resulted in a sample biased toward organizations that depend more heavily on federal funding through state entities. This study’s focus on descriptive information also cannot support findings of direct causal relationships between the infrastructure factors and decision-making around EBIs. In addition, data collection for this study took place prior to organizational changes at HHS initiated in 2025, which will affect funding provided by SAMHSA and may have ripple effects on the prevention funding infrastructure throughout the United States and down to the prevention practitioner level.

## Recommendations

This study’s findings generate some clear recommendations for prevention intervention developers and researchers. When developing interventions, including the input of prevention practitioners and funders will ensure that the interventions meet their needs across their prioritized populations and settings. In addition, developers should be intentional about overcoming the barriers faced by implementers in targeted settings, such as the barriers to working in rural settings described by NPNs in this study. Given limited funding and resources, developers also may want to consider developing lower-cost solutions and embedding their interventions in existing infrastructure, such as the example of providing opioid misuse counseling in an emergency department setting where patients are likely to receive opioid prescriptions (Bonar et al., 2021).

Study findings also have implications for disseminating those interventions. Intervention developers need to target an appropriate audience when disseminating information about interventions to encourage their adoption. This audience of decision-makers may consist of NPNs, regional entities, or community practitioners, and wide-scale adoption likely depends on inclusion of the EBI on federal or state lists or clearinghouses. When framing the pitch for an intervention, developers need to think about the needs of the identified prevention funders (audience), use terminology common to the audience’s areas of focus, and consider agency and partner priorities. Providing clear information about whether or how an EBI can be tailored to meet community needs around prevalent substance use issues (e.g., increasing concerns about polysubstance use), specific populations (e.g., by racial demographics, socioeconomic status, age, or specific risks such as experiences with homelessness), and settings would ease adoption decisions and subsequent implementation. The inclusion of substance use prevention efforts within diverse agencies also highlights the potential to leverage departmental resources and expertise to address substance use. Developers of interventions focused on populations that NPNs generally do not serve (older adults, people experiencing homelessness, and justice-involved populations) may need to look to other state agencies to disseminate their interventions.

These findings from 2023 and 2024 also point to several critical opportunities to strengthen the effectiveness, reach, and long-term sustainability of the US substance use prevention infrastructure. In their partnerships with other agencies in areas such as data sharing, the NPNs already have moved a little beyond traditional, siloed prevention models that do not sufficiently address the complexity and rapid evolution of contemporary substance use trends. However, these agencies frequently function independently, resulting in fragmented prevention efforts, gaps in local data, and missed opportunities for intervention. Encouraging more deliberate coordination among agencies could enhance the efficient use of limited state resources by embedding EBIs within the settings and communities that can most easily incorporate strategies to reach the most at-risk populations. Coordinated state infrastructures can braid diverse funding sources, guide intervention selection, provide implementation technical support, and promote shared accountability for outcomes, whereas regional intermediary organizations can provide responsiveness to local variations in risk and need.

## Conclusion

This study provides a real-time snapshot of the prevention infrastructure that shapes the funding, selection, and implementation of EBIs across states, territories, and jurisdictions in the United States. By examining the agencies and partnerships involved in prevention funding, their prioritized populations and settings, the funding pathways used, and the mechanisms guiding EBI selection, the findings can advance the overarching objective of strengthening understanding of the system among intervention developers, researchers, and funders. A clearer understanding of system functioning—including the role of SSAs, the importance of community partners, and the influence of funding pathways—can support greater collaboration and knowledge sharing, as well as more efficient adoption of prevention interventions. Ultimately, enhancing alignment across stakeholders in the intervention development and adoption process can improve the impact of EBIs and strengthen the prevention infrastructure nationwide.

## Data Availability

The data used to support the findings of this study are available on request from the corresponding author.
